# Metasurface-enabled optical encryption and steganography with enhanced information security

**DOI:** 10.1515/nanoph-2025-0015

**Published:** 2025-03-31

**Authors:** Wen Xing, Changke Bu, Xiaoyi Zhang, Duk-Yong Choi, Yang Li, Wenjing Yue, Jiaqi Cheng, Zhancheng Li, Shuqi Chen, Song Gao

**Affiliations:** School of Information Science and Engineering, Shandong Key Laboratory of Ubiquitous Intelligent Computing, 12413 University of Jinan, Jinan, 250022, China; Laser Physics Centre, Research School of Physics, Australian National University, Canberra ACT, 2601, Australia; School of Integrated Circuits, Shandong University, Jinan, 250101, China; School of Physics and TEDA Institute of Applied Physics, Nankai University, Tianjin, 300071, China

**Keywords:** metasurface, optical encryption, steganography, wavelength multiplexing, polarization multiplexing

## Abstract

Metasurfaces have attracted considerable interest in optical encryption due to their remarkable ability to manipulate light at subwavelength scales, however the aspect of encryption security remains an area requiring deeper exploration. Here, we propose and demonstrate metasurface-enabled optical encryption and steganography that provides dual-layer information protection. A secret information is embedded within multiple carrier images using a run-length encoding algorithm, dispersing the data to safeguard it against direct observation and brute-force attacks, thereby establishing the first layer of security. The second layer is achieved by encoding the multiple carrier images onto a silicon metasurface, leveraging light wavelength and polarization to generate diverse optical keys post-steganography. To validate the proposed scheme, several silicon metasurface samples are fabricated and characterized in the visible spectrum. By adjusting various combinations of optical keys, three encrypted carrier images are retrieved with high fidelity and negligible crosstalk, and the concealed secret information is successfully extracted through a corresponding decryption algorithm. The proposed approach enhances optical information security at the hardware level, making it less susceptible to leakage. It is anticipated that the demonstrated advancement will hold significant potential for applications in information security and optical anti-counterfeiting.

## 1 Introduction

Nowadays, information security is of great importance in every aspect of life. Various encryption technologies have emerged to prevent information from being stolen. Among them, optical encryption, with its unique advantages, utilizes transformations such as light interference, diffraction, and imaging to convert plaintext into complex and difficult-to-decipher optical signals, providing an efficient and reliable way for the encryption and processing of highly secure information [1], [2], [3]. With the development of new optical devices, the emergence of metasurfaces has injected new vitality into the field of modern optical encryption. Metasurfaces are artificially designed two-dimensional optical metamaterials, typically composed of arrays of nanostructures (meta-atoms) with customized shapes and subwavelength feature sizes. Due to its unprecedented manipulation capabilities over degrees of freedom (DoFs) including polarization, amplitude, phase, and orbital angular momentum of light at the sub-wavelength scale [4], [5], [6], [7], [8], [9], [10], metasurface has been successfully applied in the realization of various compact and miniaturized planar functional devices, enabling numerous applications such as hologram display [11], [12], [13], polarization conversion [14], [15], [16], [17], optical cloaking [18], [19], light focusing [20], [21], and vortex beams [22], dynamic modulation devices [23], [24], computation [25], [26], [27], and biological imaging [28], [29].

In recent years, metasurfaces have also been widely used to explore novel optical encryption by manipulating different DoFs of optical fields [30], [31], [32], [33], [34], [35], [36]. Apart from the utilization of a single DoF of light, metasurfaces have also been designed to achieve advanced optical encryption by manipulating combinations of two or more DoFs of light, aiming to increase the difficulty of information being accessed and stolen [37], [38], [39], [40], [41], [42], [43], [44], [45], [46], [47], [48], [49], [50]. Nevertheless, the information security cannot be guaranteed as the encrypted information can be directly observed once the optical key of the metasurface is stolen or subjected to brute force attacks by specific key exhaustive algorithms. Recent studies have shown that by integrating cryptographic encryption algorithms into the field of metasurface optical encryption [51], [52], [53], plaintext that is easy to be directly seen can be further transformed into incomprehensible pseudo-random ciphertext that can only be indirectly observed in specific ways. For example, common technologies such as visual secret sharing [54], computational ghost imaging [55], and one-time-pad [56] have been applied in optical encryption by taking advantage of the multidimensional manipulation of light DoFs of a metasurface. Even though these encryption schemes have improved the security level, the presence of pseudo-random ciphertext may arouse suspicion from eavesdroppers, leading to further scrutiny and attempts of decryption. Steganography, as a complementary information hiding technology with cryptography, shows prominent advantages in terms of optical encryption. Different from cryptography which mainly focuses on encryption and decryption, steganography emphasizes embedding secret information into seemingly harmless carriers through various media such as images, texts, and videos, thereby achieving the secure data transmission without arousing public suspicion [57], [58], [59], [60]. It is conceivable that applying steganography to metasurface optical encryption may open a new information protection strategy with significantly enhanced security performance. In this strategy, the carrier information will become pseudo-information with certain deceptive effect, making it difficult for unanticipated recipients to notice and extract the real information from it and effectively avoiding the risk of information leakage caused by scrutiny. Unfortunately, the importance of steganography has been underestimated and overlooked, and so far, this strategy that can both confuse eavesdroppers and ensure the secure transmission of information has not yet been reported in metasurface-based optical encryption.

**Figure 1:**
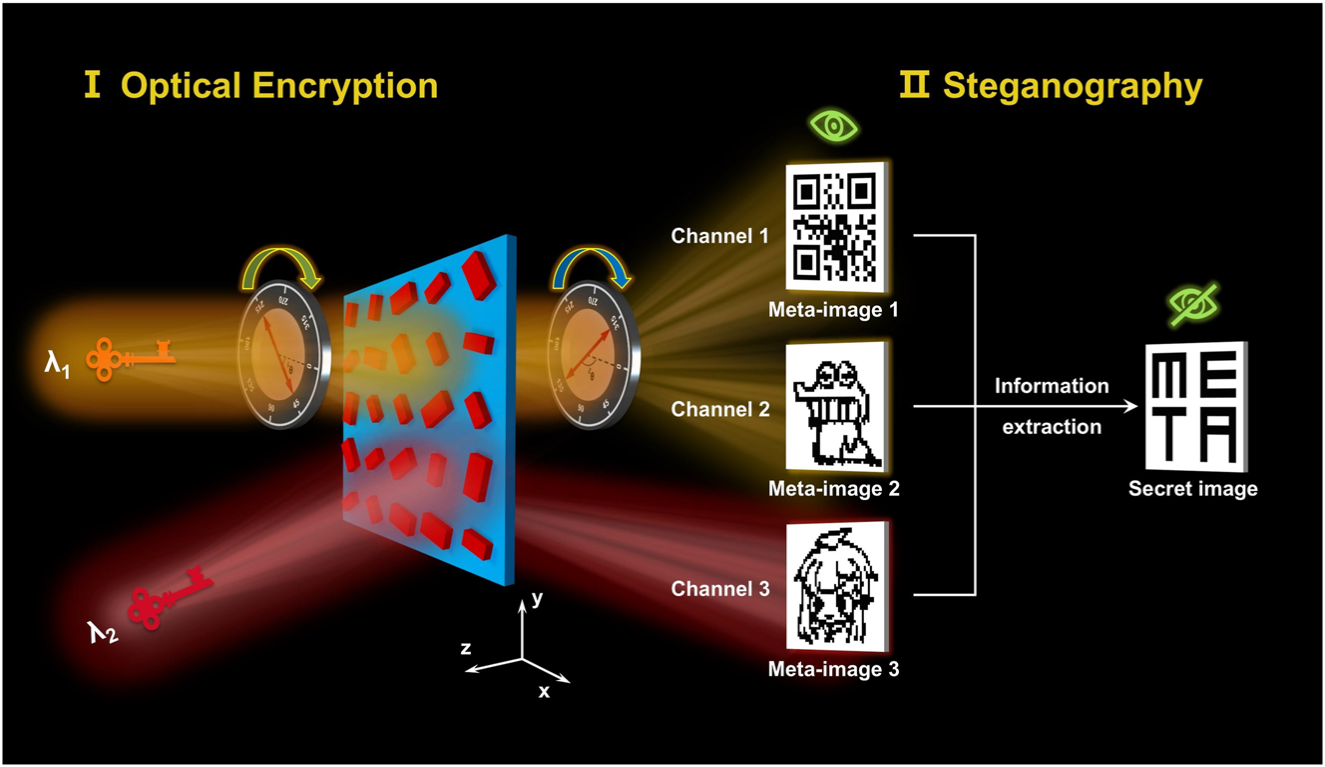
A schematic of the proposed concept of metasurface-enabled optical encryption steganography for information security enhancement. Three binary images are encoded in a single-layer metasurface in different light wavelength and polarization channels. By jointly processing the three images with a certain algorithm, the secret information can be extracted.

In this work, we propose a metasurface-enabled optical encryption steganography design scheme by combining steganography and optical encryption, aiming to achieve twofold protection of information security. As the first security barrier, a steganography algorithm based on run-length encoding (RLE) is utilized to dispersedly embed the secret information into multiple carrier images. This results in observers only being able to visually perceive the surface information presented in the carrier images, thereby protecting the secret information from direct observation. The multiple carrier images after steganography are further encoded into the multiplexed metasurface for encryption. The final transmitted information can only be extracted by decrypting the optical images hidden in all the channels. As the second security barrier, different keys are deliberately induced for decrypting the optical images hidden in the metasurface. To maximize the differentiation of keys for each encrypted image, various optical parameters are introduced, ensuring that the keys for different images have significant differences in levels. The scheme is specifically validated by a wavelength and polarization multiplexing metasurface, and the process of decrypting and extracting the real information from multiple images is schematically shown in Figure 1, where three binary images can be decrypted from the metasurface by adjusting different key combinations of light wavelength and polarization. Specific algorithms are further used to extract and recover the secret information hidden in multiple images. The proposed scheme is successfully validated through a dual-wavelength and three-channel silicon metasurface, both numerically and experimentally. Our work realizes the integration of steganography with optical encryption on a physical hardware level, which provides an interesting approach for information encryption or hiding and will be of interest in fields of information security and optical anticounterfeiting.

## 2 Results and discussion

### 2.1 Optical encryption metasurface with wavelength and polarization multiplexing

**Figure 2:**
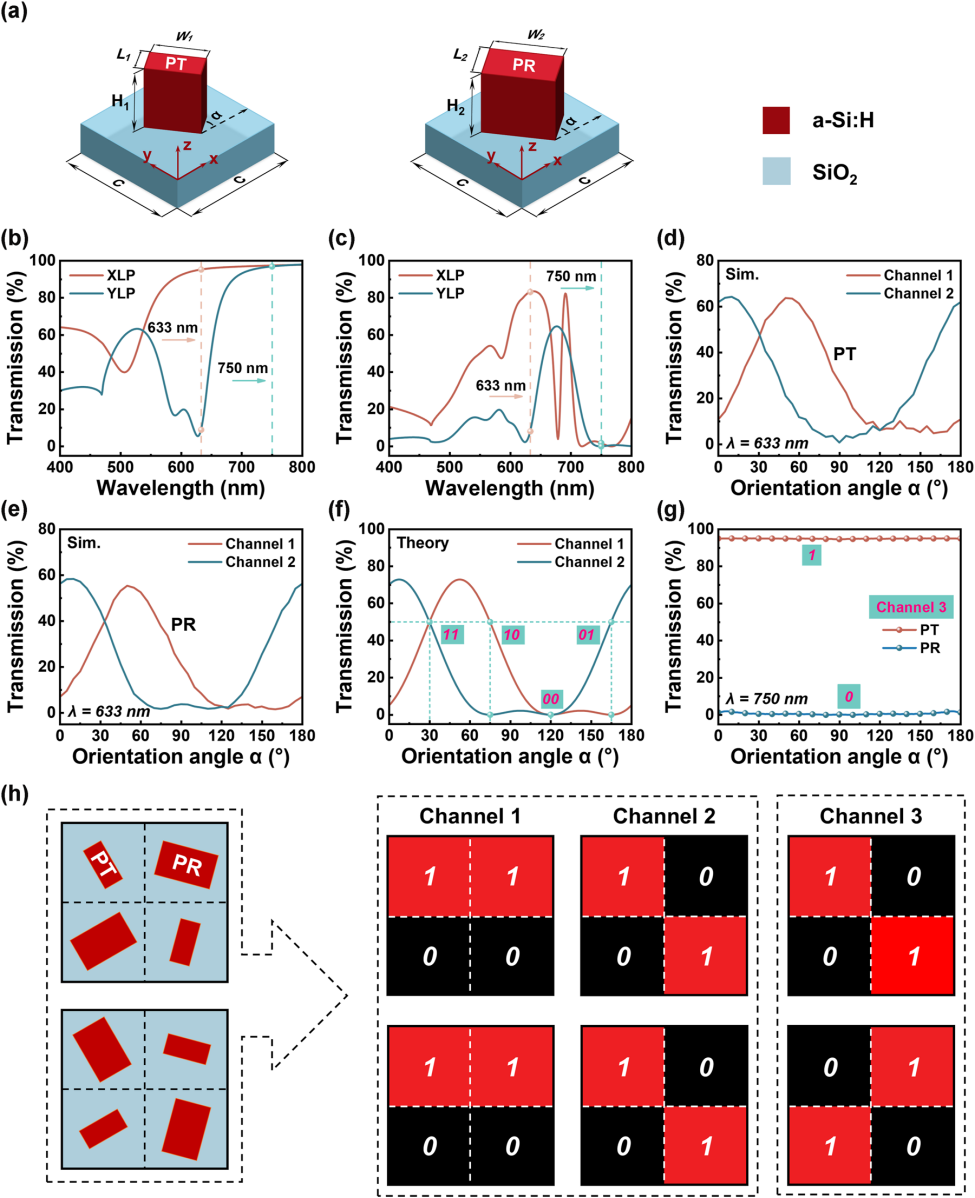
Numerical simulation results of the selected nanostructures featuring three-channel binary encoding capability. (a) Schematic diagram of the metasurface unit cells of PT and PR. Simulated polarized transmission spectra of the non-rotated (b) PT and (c) PR. Simulated transmission as a function of the orientation angles of (d) PT and (e) PR in Channel 1 and Channel 2 at the wavelength of 633 nm. (f) Theoretical transmission as a function of the ideal polarizer orientation angle in Channel 1 and Channel 2, with dashed lines indicating the four selected orientation angles for realizing 2-bit binary intensity encoding. (g) Simulated total transmission of the TP and TR with different nanopillar orientation angles at wavelength of 750 nm. (h) Illustration of recording three-channel binary intensity encoding states into two types of nanopillars with different orientation angles.

As the first step, to achieve independent encoding of three images at different security levels on a single platform, a metasurface with wavelength- and polarization-selective amplitude manipulation ability is proposed. The metasurface unit cell is composed of a nanopillar placed on top of a SiO_2_ substrate. The nanopillar is made of hydrogenated amorphous silicon (a-Si:H) considering its high refractive index and low absorption at the selected two operating wavelengths, *λ*_1_ = 633 nm and *λ*_2_ = 750 nm. Specifically, two nanopillars with distinct geometric sizes are incorporated for subsequent implementation of the optical encryption and steganography. The two nanopillars are anticipated to concurrently function as the linear polarizer at wavelength *λ*_1_, while they respectively exhibit polarization-independent high transmission and reflection at wavelength *λ*_2_ [48]. For convenience, hereafter the two nanopillars are named as PT and PR, respectively, where the first letter “P” indicates the polarizer function at *λ*_1_, while the second letter “T” and “R” represent the high transmission and high reflection at *λ*_2_, respectively, guaranteeing polarization-independent high transmission difference at *λ*_2_. Numerical simulations based on the finite-difference time-domain method are conducted to find the proper nanopillars that meet our requirements and detailed simulation results can be found in Section S1 in Supplementary Material. As shown in Figure 2a, the specific periodicity of the metasurface unit cell (C) is 320 nm. Both PT and PR are designed to be 220 nm in thickness, while their geometric sizes (*L*_1_, *W*_1_) and (*L*_2_, *W*_2_) are (60 nm, 130 nm) and (140 nm, 225 nm), respectively. For realizing the three-channel binary images, the rotation angle (α) of PT and PR become critical. Since PT and PR both exhibit different geometric sizes along their own two axes, they will exhibit diverse polarization-dependent transmission spectra in principle. Figure 2b and c respectively display the simulated transmission spectra (400–800 nm) of the non-rotated PT and PR under incidence of x-polarization (XLP) and y-polarization (YLP). At the wavelength of 633 nm, the maximum transmission efficiencies of the PT and PR are 95 % and 83 %, respectively, with the transmission differences between XLP and YLP for PT and PR being approximately 87 % and 75 %. At the wavelength of 750 nm, the transmission efficiencies under XLP and YLP incidences are both above 94 % for PT, while they are below 2 % for the PR. Note that the transmission efficiencies shown in Figure S1 in Supplementary Material, in the regions surrounding the selected PT and PR, are similar, indicating that slight variations in structural dimensions will not significantly impact the desired metasurface functionalities and the quality of the meta-images, thereby ensuring that the proposed scheme remains robust against structural inaccuracies. Given a fixed incidence polarization, the output light intensity from a nano-polarizer can be modulated by altering its orientation angle, which is mathematically described below. It is known that the Jones matrix M_α_ of a nanopillar with an in-plane orientation angle of *α* can be expressed as:

(1) $$\begin{array}{ll} {\text{M}}_{\alpha } & =\text{R}\left(\alpha \right)\cdot {\text{M}}_{0}\cdot \text{R}\left(-\alpha \right)\\ & =\left[\begin{array}{cc} \cos\alpha & -\sin\alpha \\ \sin\alpha & \cos\alpha \end{array}\right]\left[\begin{array}{cc} \text{A} & 0\\ 0 & \text{B} \end{array}\right]\left[\begin{array}{cc} \cos\alpha & \sin\alpha \\ -\sin\alpha & \cos\alpha \end{array}\right] \end{array}$$

where R(α) is the rotation matrix, M_0_ denotes the Jones matrix of an ideal nanopillar without cross-polarization components, and A and B are the complex transmission (or reflection) coefficients of light polarized along the x- and y-axis, respectively. In addition to the nanopillar, a polarizer and an analyzer are also employed to select the polarization component of the incident and transmitted light in our design. Upon incidence of a linearly polarized light (Jones vector J_0_) with polarization angle θ_1_, the light transmitting through the metasurface (M_α_) and passing through the analyzer (M_θ_) can be finally expressed as:

(2) $$\begin{array}{ll} {\text{J}}_{1} & ={\text{M}}_{\theta }\cdot {\text{M}}_{\alpha }\cdot {\text{J}}_{0}\\ & =\left[\begin{array}{cc} cos^{2}\theta_{2} & \sin\theta_{2}\cos\theta_{2}\\ \sin\theta_{2}\cos\theta_{2} & sin^{2}\theta_{2} \end{array}\right]M_{\alpha }\left[\begin{array}{c} \cos\theta_{1}\\ \sin\theta_{1} \end{array}\right] \end{array}$$

where M_θ_ is the Jones matrix of the analyzer, θ_1_ and θ_2_ are the transmission axis directions of the polarizer and analyzer, respectively. Given that the intensity of the linearly polarized light after the polarizer is I_0_, the output light passing through the final analyzer is (see more details in Section S2 in Supplementary Material):

(3) $${\text{I}}_{1}={\text{I}}_{0}{\left[\frac{\text{A}-\text{B}}{2}\cos\left(2\alpha -\theta_{2}-\theta_{1}\right)+\frac{\text{A}+\text{B}}{2}\cos\left(\theta_{2}-\theta_{1}\right)\right]}^{2}$$

For an ideal polarizer (i.e., A = 1, B = 0), Equation (3) can be simplified to:

(4) $${\text{I}}_{1}=\frac{1}{4}{\text{I}}_{0}{\left[\cos\left(2\alpha -\theta_{2}-\theta_{1}\right)+\cos\left(\theta_{2}-\theta_{1}\right)\right]}^{2}$$

The above equation clearly shows that the output light intensity can be customized by α, θ_1_ and θ_2_. Furthermore, a 2-bit or dual-channel binary intensity design can be readily achieved by elaborately selecting two non-orthogonal polarized light paths. As an example, channel 1 is set to θ_1_ = 30° and θ_2_ = 75°, and θ_1_ = −15° and θ_2_ = 30° for channel 2. The transmission of PT and PR under the above two channels are respectively simulated as a function of their orientation angles for wavelength of 633 nm, as depicted in Figure 2d and e. Figure 2f further presents the theoretical dual-channel transmission intensity distributions to intuitively compare the intensity tuning performance of the designed nano-polarizers and the ideal polarizers, where a good agreement can be found between them. Subsequently, four orientation angles (30°, 75°, 120° and 165°) with high and/or low transmission at the two channels, corresponding to four different binary coding states of “**11**”, “**10**”, “**00**”, and “**01**”, are selected for later use of encoding two binary images. For wavelength of 750 nm, the total transmission of PT (PR) maintains stably at a high (low) level with respect to different orientation angles as witnessed in Figure 2g. This feature (transmission intensities invariant to orientation angle and incidence polarization) further enables another 1-bit encoding of the light intensity (PT as “**1**” and PR as “**0**”). Accordingly, a third binary image that is only related to the nanopillar size can be designed at this wavelength (defined as channel 3). In short, the binary image in channel 3 determines which type of nanopillar is needed, while the other two binary images in channel 1 and channel 2 govern the orientation angle of the nanopillar. Figure 2h shows a schematic of how the three-channel light intensity distributions are affected by the arrangement of the nanopillars. It should be noted that such three-channel binary intensity encoding method is only valid in a relatively narrower wavelength range. A discussion on the operation wavelength range of the proposed metasurface can be found in Section S3 in Supplementary Material.

### 2.2 Secret information embedding process based on multi-carrier steganography algorithm

**Figure 3:**
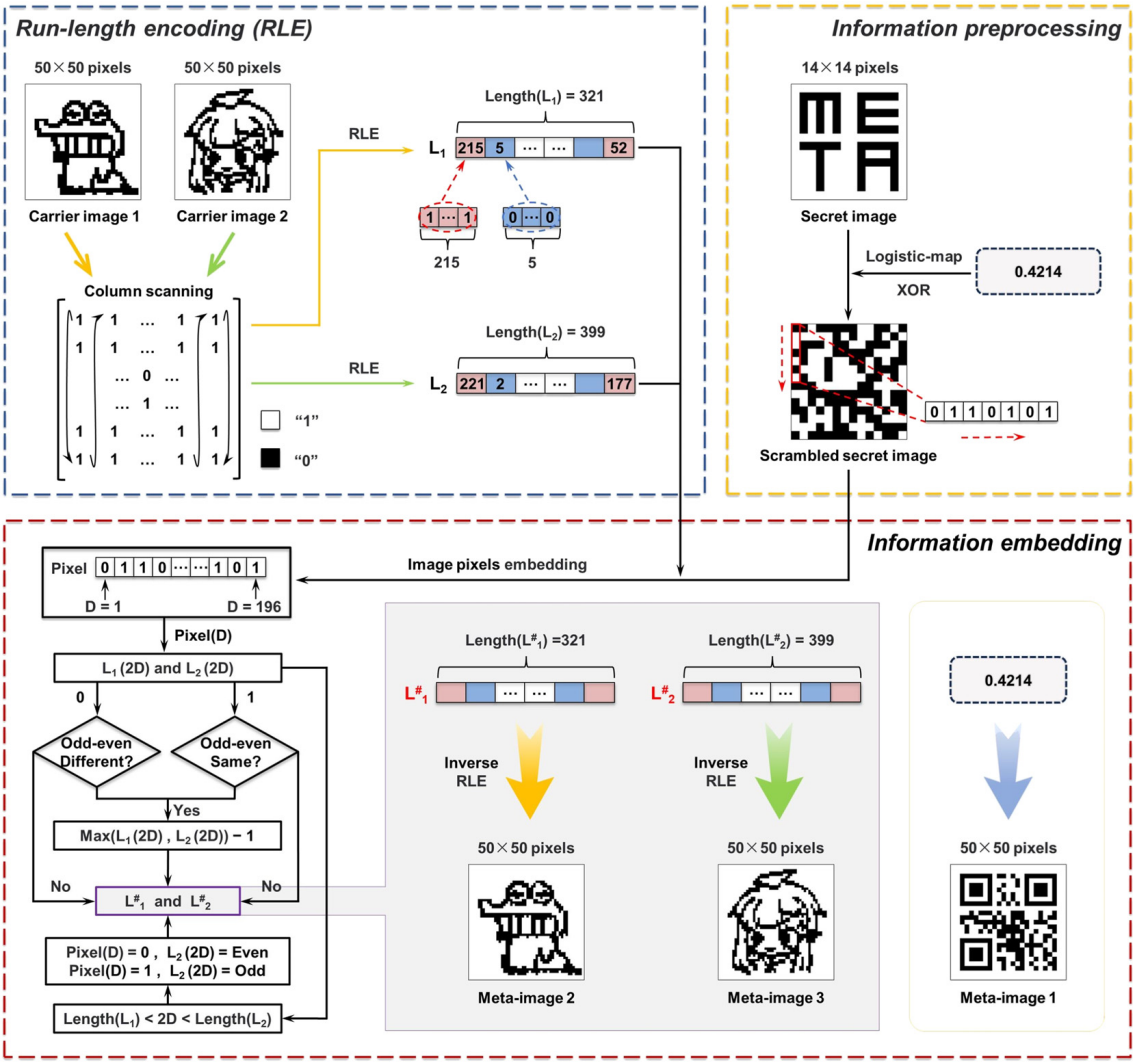
Process of embedding a secret information in multiple carrier images. The dashed blue box indicates the RLE process for encoding the carrier images. The orange box represents the pre-processing the secret information. The red box illustrates the process of embedding the information into multiple carrier images according to algorithm.

Based on the selected PT and PR and the abovementioned design principles, it is feasible to encode three images into a single-layer metasurface, providing an optical encryption barrier for the carrier image after steganography. Moreover, as a proof of concept for multi-carrier steganography, the steganography algorithm of RLE which resorts to the parity of optical image pixels to embed more confidential secret information on top of the optical encryption level, is further introduced and the process is illustrated in Figure 3. The RLE is considered a popular compression technique [61], [62]. In RLE, when characters in a string appear consecutively, they are replaced by the number of times they occur, which is referred to as the run-length value. Since binary images contain numerous consecutive identical blocks of black and white pixels, they can also be processed by RLE. We select two carrier images (image 1: cartoon animal; image 2: cartoon girl) with 50 × 50 pixels each, and encode their pixels using RLE, as shown by the dashed blue box in Figure 3. For carrier image 1, it can be considered as a 50 × 50 matrix, where white pixels are represented as 1 and black pixels 0. Then, it can be scanned column-by-column to transform the dimensions into a consecutive 01 sequence. Subsequently, the 01 sequence is processed through RLE, whereby consecutive black and white pixels are replaced with their respective counts of occurrences. All the run-length values after encoding form a run-length sequence (named as L_1_), whose length is denoted as Length (L_1_). Based on our selected carrier image 1, the first run-length value in the sequence is 215, indicating 215 consecutive occurrences of 1, corresponding to 215 consecutive white pixels. Similarly, the sequence formed by encoding carrier image 2 using RLE is named as L_2_, and its length is Length (L_2_).

As the carrier images are binary images, any information to be embedded within them needs to be converted into a binary sequence. In our design, a 14 × 14 pixels binary image displaying the content “META” is our final transmitted secret information for hiding. Due to the presence of consecutive identical pixels in the secret information, a logistic map is used to generate a chaotic iterative sequence with non-periodic and non-convergence properties for scrambling preprocessing to avoid regularity and further enhance the security of the information, with the expression as follows:

(5) $${\text{x}}_{\text{n}+1}=\mu {\text{x}}_{\text{n}}\left(1-{\text{x}}_{\text{n}}\right)$$

where x_n_ represents the state value at the n-th iteration (x_n_ ∈ (0,1)), x_n+1_ is the next state value of x_n_, and *μ* is the control parameter (μ ∈ (0,4)) [51], [61], [63]. When *μ* is closer to 4, the generated iterative sequence has the chaotic properties described above and can be used to realize information encryption. Since the domain of the resulting iterative sequence lies within [0, 1], the threshold is defined as the initial value x_0_ of the logistic map to construct a 14 × 14 binary matrix for an exclusive OR (XOR) operation with the secret information. Then, the 01 sequence (Pixel) to be embedded can be obtained by scanning the scrambled secret information column by column, as depicted by the orange box in Figure 3. Next, according to certain algorithmic rules, the information from the sequence is dispersedly embedded bitwise into L_1_ and L_2_, as shown by the red box in Figure 3 (D represents the position of each binary number in the sequence Pixel). During embedding, the run-length values in L_1_ and L_2_ corresponding to the same even position (run-length values of black pixel) are first detected. When the embedding information bit is 0, the larger value of the two values is modified (minus 1) to ensure they have the same parity. Conversely, when the information bit is 1, similar modifications are made to make them have different parity (if the two run-length values have the same size at this point, the modification is made to one of the two sequences). When the embedding position in the run-length sequence exceeds the length of L_1_ (L_2_), the remaining information is embedded solely in L_2_ (L_1_). In this case, if the information bit is 0, run-length value at the corresponding position in L_2_ (L_1_) is modified to ensure it becomes even; conversely, it is modified to become odd. When the parity matches the value to be embedded, no modification is made. It is worth noting that when a run-length value is modified, the modification is made to the last bit of the consecutive identical binary number in its unencoded state, changing it from 0 to 1, corresponding to changing the pixel from black to white in the carrier image. Following the above rules, the secret information can be successfully embedded into two run-length sequences, and the sequences after embedding the information are named as L^#^_1_ and L^#^_2_, respectively. Then, the inverse process of RLE is performed on each sequence, decoding the run-length values to recover consecutive black and white pixels, thereby reconstructing two carrier images with hidden information, each of which is 50 × 50 pixels. Meanwhile, a string of “0.4214” is encoded into a QR code (wherein, 4 represents the value of *μ* in the mapping formula; 2 the prompt information of the embedding location; 14 the pixel size of the secret image information; the entire “0.4214” the initial value x_0_ of the logistic map) for transmitting parameter information related to the extraction and recovery of the secret information. The three images (the QR code, the reconstructed cartoon animal, and the reconstructed cartoon girl) are designated as meta-image 1, 2, and 3, respectively. It is worth noting that the changes of some pixels are visible to the naked eye on the meta-image 2 and 3 after embedding the information. This is due to the small size of the pixels in the chosen carrier images, which is also an indicator of the difference between the carrier images before and after information embedding.

### 2.3 The simulation and experiment results of optical encryption metasurface

**Figure 4:**
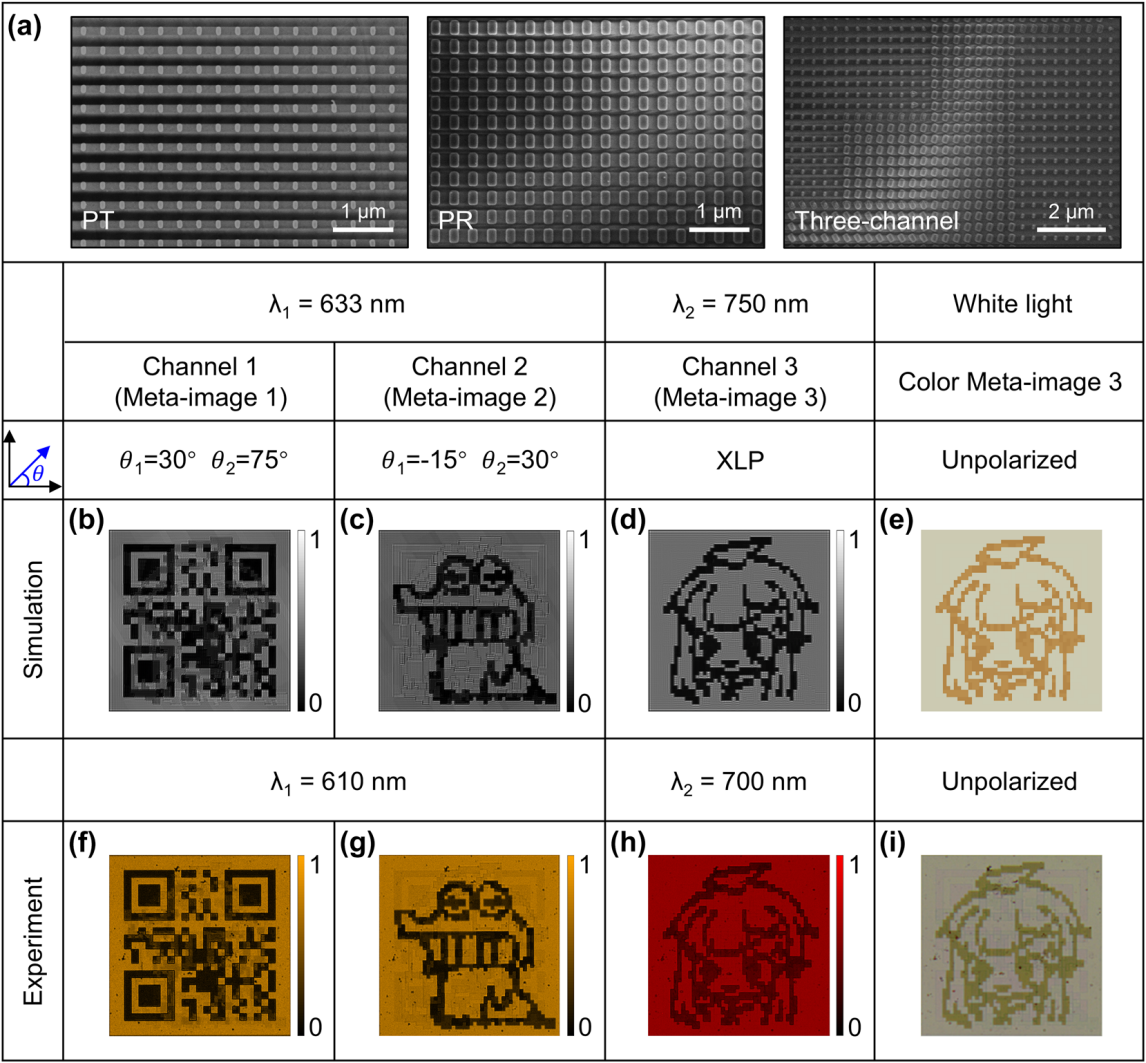
Simulation and experimental results of the metasurface. (a) SEM image of the fabricated metasurface samples. Simulated light intensity distributions in (b) Channel 1 and (c) Channel 2 at wavelength of 633 nm, and (d) Channel 3 at wavelength of 750 nm under XLP, as well as (e) under unpolarized white light incidence. Corresponding experimental results for (f) Channel 1 and (g) Channel 2 at wavelength of 610 nm, and (h) Channel 3 at wavelength of 700 nm, as well as (i) unpolarized white light incidence.

To experimentally verify the proposed idea, three a-Si:H metasurface samples are simultaneously fabricated on the same silica substrate using the standard electron beam lithography technique, and their scanning electron microscope (SEM) images are shown in Figure 4a. Detailed fabrication procedure can be found in the Experimental Section. Two metasurface samples (denoted as PT and PR in Figure 4(a)), with each occupying an area of 32 × 32 μm^2^ containing only non-rotated PT and PR, are used to find the two practical operating wavelengths. The fabricated PT and PR samples are characterized by a spectrometer that is fiber-coupled to a microscope (details are in the Experimental Section) and the measured polarized transmission spectra are presented in Section S4 in Supplementary Material, which clearly show the polarization-dependent properties. Despite the slight shift of the resonant wavelengths, the transmission spectra in measurement comply well with those in simulation. Based on the measured spectra, we designate 610 nm as the wavelength where the fabricated PT and PR both function as the nano-polarizer, and 700 nm is selected as the wavelength where polarization-independent high transmission difference between the PT and PR is realized. Next, the feasibility of the proposed metasurface-enabled optical encryption steganography for information security enhancement strategy is verified by the three-channel sample which is designed through carefully selecting the nanopillars and setting their spatial orientation angle based on the previously discussed metasurface design rule and the aforementioned three meta-images. To be specific, the three-channel sample contains 50 × 50 pixels, with each pixel containing 8 × 8 nanopillars, leading to a total metasurface area of 128 × 128 μm^2^. The concept of integrated optical encryption and steganography is first validated by conducing numerical simulations to extract the three binary images hidden in the metasurface. Considering the limited computing resources, the size of the metasurface in simulation is reduced to 64 × 64 μm^2^, with each pixel including 4 × 4 nanopillars, and more simulation details are provided in Section S5 in Supplementary Material. Figures 4b through 4d provide the simulated results under the designed three channels. At the wavelength of 633 nm, an optical polarizer and an analyzer are needed and set to the correct polarization angle for decoding the embedded meta-images, corresponding to a high encryption security level. Two meta-images displaying a QR code and a cartoon animal (Figure 4b and c) are successfully obtained with low crosstalk under the designed two non-orthogonal polarized light paths (i.e., meta-image 1: θ_1_ = 30° and θ_2_ = 75°, and meta-image 2: θ_1_ = −15° and θ_2_ = 30°). Conversely, for the low encryption security level at wavelength of 750 nm, the meta-image 3 of the cartoon girl can be revealed under arbitrarily polarized light incidences. Here, we present the results under the incidence of XLP (Figure 4d), while the results under other polarization incidences can be seen Section S6 in Supplementary Material. Additionally, since PT and PR exhibit different transmission spectra, they are likely to appear in different colors under white light illumination, which can be roughly estimated by averaging the polarized transmission spectra. Figure 4e displays the calculated light intensity distribution for incidence of unpolarized white light, where a colored cartoon girl can also be revealed. Corresponding to the simulation results, Figure 4f through 4h show the transmission intensity profiles that are experimentally obtained at the wavelengths of 610 nm and 700 nm by placing the corresponding optical narrowband filters in the optical path. All expected three-channel meta-images of QR code, cartoon animal, and cartoon girl are vividly observed with high fidelity and negligible crosstalk. A quantitative analysis of the crosstalk level can be found in Section S7 in Supplementary Material. Figure 4i shows the experimentally obtained transmission intensity distribution under white light incidence, consistent with the simulation result. It should be noted that although the meta-image 3 can also be distinguished by color under white light, this does not pose a risk of leakage to the hidden information, as all the meta-images themselves are pseudo-information with certain confusing effect, and observation of only the meta-image 3 cannot reveal the secret information. On the other hand, to prevent the meta-image in Channel 3 from being observable to the naked eye, one may consider to redesign and optimize the two nanostructures to have identical transmission spectra in the visible range [64], by shifting the working wavelengths – where the metasurface acts as a linear polarizer and polarization-independent transmitter and reflector – into the near- or mid-infrared wavelengths.

### 2.4 Secret information extraction and recovery process

**Figure 5:**
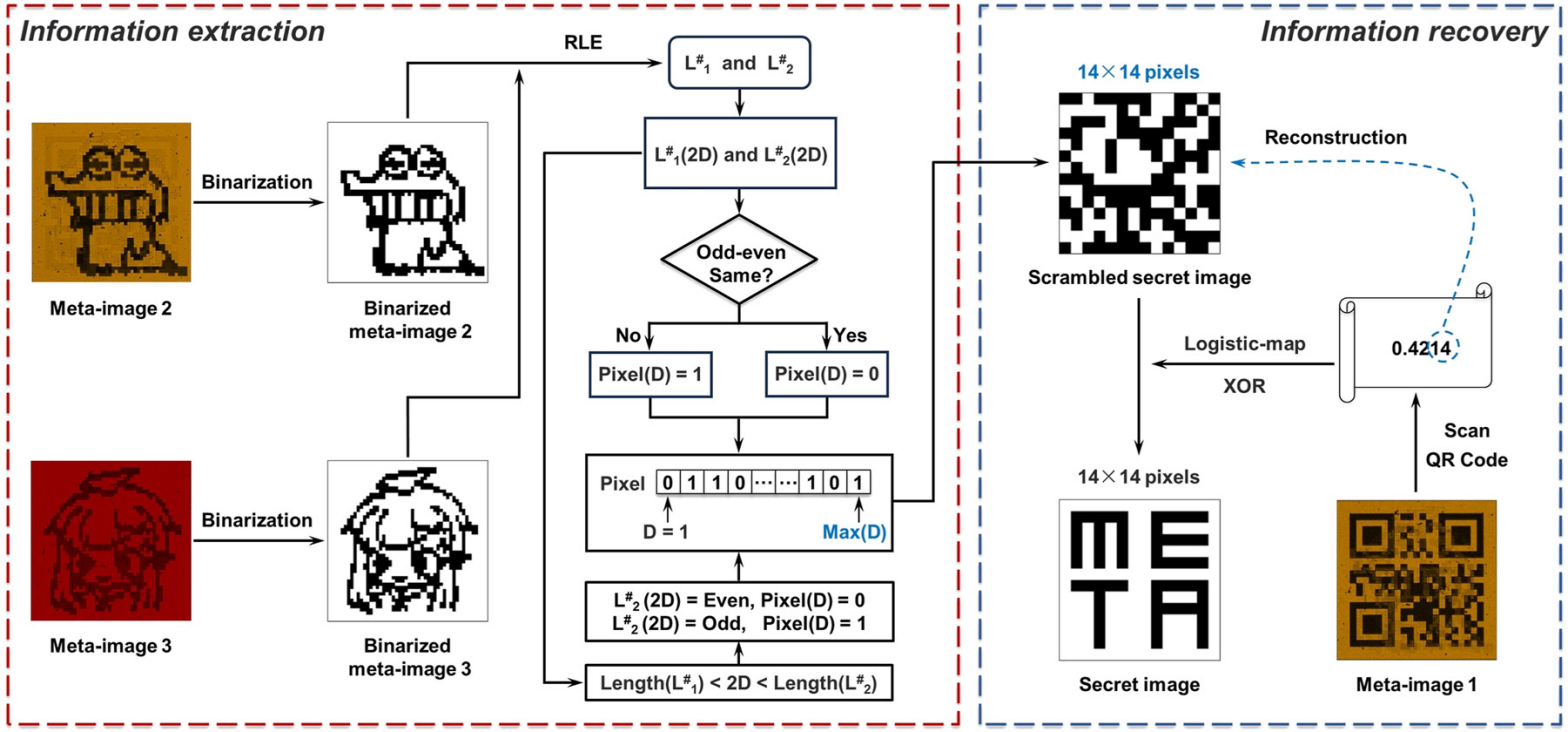
Process of extracting and recovering secret information. The red box shows the process of extracting information from meta-images obtained from the metasurface. The blue box demonstrates the recovery process of information.

The final transmitted secret information hidden in the single-layer metasurface can be decrypted through two steps. Firstly, the different optical keys are used to extract all nano-scale meta-images (as have been done in Figure 4), which is the basis for accurate information extraction. Secondly, the correct decryption algorithm is utilized to extract and recover the secret information. The process of information extraction and recovery is shown in Figure 5. A decryptor initially obtains a parameter (the string “0.4214”) for information extraction and recovery by scanning meta-image 1 (QR code), which is crucial for accurately retrieving the secret information. Here, the parameter information is directly obtained by scanning the QR code, but the obtained parameter will not easily suggest its significance to observers other than the intended recipient, reminding them of the information hidden in the grayscale images, thereby arousing suspicion, and prompting further scrutiny from eavesdroppers. It is worth mentioning that, for enhanced security, QR codes can be a way for the link sender to receiver, enabling the decryptor to obtain parameters after authentication. However, as part of the secret information extraction process, the decryptor needs to perform binarization and nearest interpolation on the obtained meta-images 2 and 3 respectively, and then the run-length sequences L^#^_1_ and L^#^_2_ of the two images are obtained by RLE. Next, the parity of the run-length values at corresponding even position in the two sequences is compared simultaneously. When the parity is the same, the extracted binary bit at that position is 0; otherwise, it is 1. If the extraction position exceeds the length of L^#^_1_ (L^#^_2_), the information will only be extracted from sequence L^#^_2_ (L^#^_1_). The specific rule is that when the run-length value at the extraction position in L^#^_2_ (L^#^_1_) is even, the extracted binary bit at that position is 0; otherwise, it is 1. The information extraction process is shown in the red box in Figure 5. The hidden scrambled secret image is reconstructed after extracting all embedded information, and the secret image information can be recovered by performing XOR logic operation with the iterative sequence generated from the parameter 0.4214, as shown in the blue box in Figure 5. To facilitate the visualization of the above information extraction and recovery process, a graphic user interface is developed in MATLAB. By setting the parameter obtained from channel 1 and importing the experimentally obtained meta-images information of channel 2 and channel 3, the concealed secret information (META) is accurately extracted through three steps of control. Details can be found in Video S1 in the Supplementary Material.

The three meta-images play equally important roles in the final information extraction and recovery process. If any one of these images is not correctly obtained during the metasurface decryption stage, it will be impossible to extract the secret information ultimately. As the proof that the secret information can be correctly extracted only by using the designed meta-images, the erroneous result by attempting to extract the information with other undesigned images is also presented. In addition, even with the correct meta-images, the embedded secret information still cannot be extracted with an incorrect input parameter in Channel 1. Video S2 in the Supplementary Material clearly shows that no meaningful information can be extracted under the abovementioned conditions. Interestingly, as the steganography algorithm operates on the binary data, our design can not only hide image information but also hide textual information. To demonstrate the versatility of the carrier images and steganography algorithm, the carrier images used in the above design are still being used to design two metasurfaces (metasurface B and metasurface C), in which the hidden information is two textual information expressed in Chinese and English respectively (see Section S8 in Supplementary Material). Additionally, we conduct an analysis of errors that may occur during the information extraction and recovery process (Section S9 in Supplementary Material). As RLE is a lossless encoding method, this pixel-sensitive method of information hiding can effectively prevent brute force attacks and facilitate the destruction of secret information in secure transmission applications.

Despite our scheme highlight the potential of metasurface-based encryption for secure information hiding, it is important to acknowledge that, at the current stage, the practical application of the proposed metasurface-based encryption schemes may face challenges in terms of scalability and replicability. For instance, the high cost and complexity of nanofabrication techniques could limit their widespread adoption, while the deterministic nature of metasurface designs may render them susceptible to replication by sophisticated counterfeiters. As a result, the approach may be effective in niche applications with moderate counterfeiting risks. However, these challenges could be addressed in the future through advancements in cost-effective nanomanufacturing (e.g., nanoimprint lithography) and the integration of multi-layer security features (e.g., dynamic optical properties, quantum dots, or biometric markers). A detailed discussion on further increasing the information channels and enhancing the security can be found in Section S10 in Supplementary Material, and a qualitative analysis of the encryption complexity is provided in Section S11 in Supplementary Material. Lastly, although our method uniquely treats optically encrypted images as pseudo-information, concealing real information within pixels via an algorithmic process and thereby enhancing security and deception, it may face pixel sensitivity issues due to its reliance on the integrity of the carrier images.

## 3 Conclusions

In summary, we have proposed and experimentally demonstrated a metasurface-enabled optical encryption steganography design scheme for information security enhancement. In the scheme, the multiple images to be embedded in the metasurface partly serve as the pseudo-information, while a steganography algorithm based on RLE was specially introduced when designing the meta-images. As a result, an extra secret information can be safely written into the metasurface. For practical validation of the scheme, silicon meta-atoms were systematically investigated in terms of its amplitude responses at two different visible wavelengths. With the help of the two appropriate a-Si:H nanopillars, along with the engineering of their orientation angles, wavelength- and polarization-selective amplitude manipulations were achieved and made possible the encryption of three binary images on a single metasurface platform. Three metasurface samples were fabricated to experimentally validate the proposed idea. With proper setting of the correct optical keys, the designed three nanoscale binary images were successfully captured, and consequently the hidden secret information of steganography was accurately retrieved. As such, our work offers an intriguing approach for optical information hiding or anti-counterfeiting, making information more secure and less susceptible to be leaked.

## 4 Experimental section

### 4.1 Sample fabrication

The samples were fabricated using a series of standard processes including electron beam lithography (EBL), Al etch mask liftoff, and silicon plasma etching. Firstly, hydrogenated amorphous silicon (a-Si:H) film was deposited using plasma-enhanced chemical vapor deposition (Plasmalab 100 from Oxford) on fused silica (SiO_2_) substrate. Next, a positive electron resist (ZEP520A from Zeon Chemicals) was spin-coated onto the film. The designed metasurface pattern was then written on the resist using an electron beam writer (EBL, Elionix Boden 125), and accompanied by the development in ZED-N50. Subsequently, an aluminum film was deposited via electron-beam evaporation (Temescal BJD-2000) on the substrate, and it was patterned by lifting off the resist using a solvent (ZDMAC from Zeon Co.). The patterned aluminum was utilized as a hard mask during dry etching, thereby transferring the designed pattern to the underlying a-Si:H layer through fluorine-based inductively-coupled-plasma reactive ion etching (Oxford Plasmalab System 100). The residual aluminum from the patterned nanopillars was etched in phosphoric/nitric/acetic acids mixed solution.

### 4.2 Optical characterization

An integrated optical microscope (“BX53M”, Olympus) is used for characterizing the fabricated metasurface samples. For the transmission spectra measurement, the light emitting from the white light source was first passed through a polarizer and slightly focused on the metasurface sample using a lens. The light was then transmitted through an objective lens (MPlanFL N 20X/0.45, Olympus), followed by an in-built beam splitter, which directed the light to a fiber (“QP600-2-SR, Ocean Insight”) coupled spectrometer (“Maya2000 Pro”, Ocean Insight) for measuring the transmission spectrum and to a CCD camera (“SC180”, Olympus) for recording the transmission intensity pattern at the sample surface. Particularly for the intensity pattern measurement, an optical filter (BPH610-10 nm, BPH700-10 nm, Rayan Optics) was selectively inserted before the metasurface sample, while an analyzer was placed between the objective lens and the beam splitter for selecting the polarization component of the output light. The experimental setup can be seen in Section S12 in Supplementary Material.

## Supplementary Material

Supplementary Material Details
