# Supplementary material for: Metasurface-enabled optical encryption and steganography with enhanced information security
Source: Nanophotonics. 2025 Mar 31;14(9):1391–403. doi: 10.1515/nanoph-2025-0015 (PMC12038608; doi:10.1515/nanoph-2025-0015)
Supplement: Supplementary file 1 — Supplementary Material Details [file j_nanoph-2025-0015_suppl_001.pdf]

Supplementary Material

Metasurface-Enabled Optical Encryption and Steganography with Enhanced Information Security

Wen Xing[1], *Changke Bu*[1], *Xiaoyi Zhang*[1], *Duk-Yong Choi*[2], *Yang Li*[3], *Wenjing Yue*[1*], *Jiaqi Cheng*[4], *Zhancheng Li*[4], *Shuqi Chen*[4*], *Song Gao*[1*]

[1]School of Information Science and Engineering, Shandong Key Laboratory of Ubiquitous Intelligent Computing, University of Jinan, Jinan 250022, China

[2]Laser Physics Centre, Research School of Physics, Australian National University, Canberra ACT 2601, Australia

[3]School of Integrated Circuits, Shandong University, Jinan 250101, China

[4]School of Physics and TEDA Institute of Applied Physics, Nankai University, Tianjin 300071, China

S1. Polarized transmission efficiency as a function of nanopillar lateral dimensions at dual wavelengths

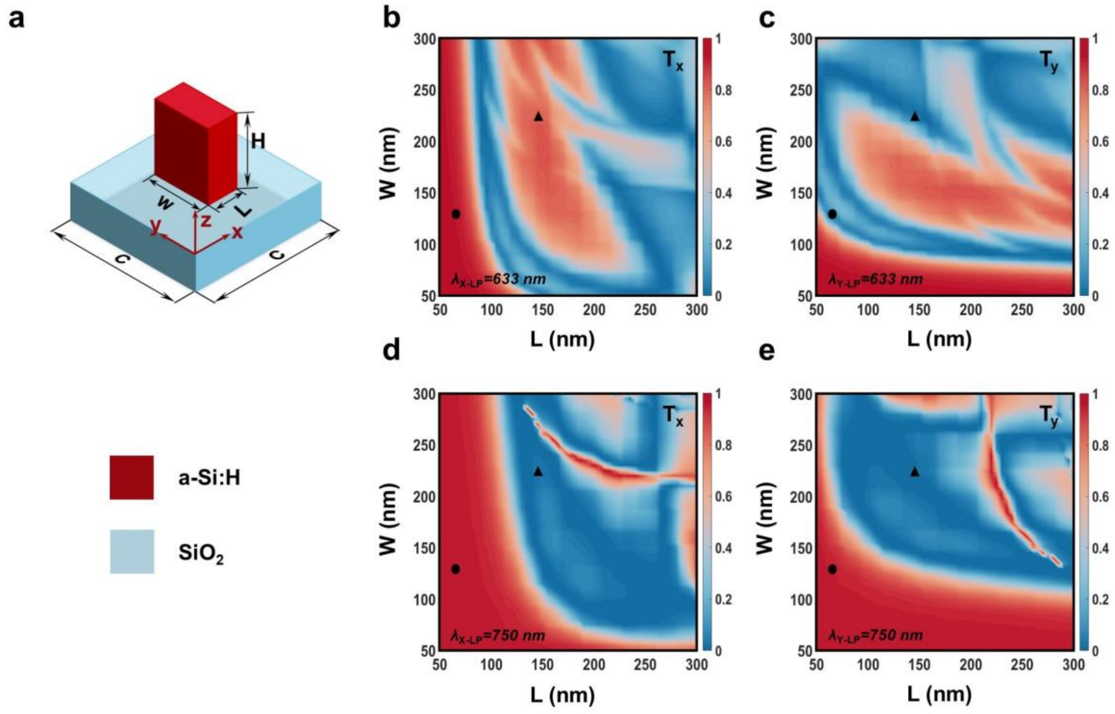

Figure S1. (a) Schematic of the metasurface unit cell, in which an a-Si:H nanopillar is placed on a SiO_2 substrate. Transmission efficiencies as a function of nanopillar lateral dimension under (b) XLP and (c) YLP incidences at wavelength of 633 nm. Transmission efficiencies as a function of nanopillar lateral dimension under (d) XLP and (e) YLP incidences at wavelength of 750 nm.

Figure S1(a) illustrates the schematic diagram of the designed metasurface unit cell, where a rectangular nanopillar of hydrogenated amorphous silicon (a-Si:H) is placed on a silicon dioxide (SiO_2) substrate. The period of the unit cell is C=320 nm, which is approximately half of the operating wavelengths, effectively avoiding higher-order diffraction. To ensure that the desired nanopillars exhibit excellent optical responses at the operating wavelength of λ_2 without compromising the functionality at wavelength of λ_1, the height of the nanopillar is optimized to be H=220 nm. Figures S1(b) and S1(c) show the swept transmission efficiencies as a function of the nanopillar length (L) and width (W) under the incidence of XLP and YLP at the wavelength of λ_1, respectively. Two nanopillars marked in solid circle and triangle show that they exhibit a significant difference in transmission between the long and short axes. Similarly, the transmission efficiencies at wavelength of λ_2 are shown in Figures S1(d) and S1(e). One of the selected two nanopillars endows high transmission for both XLP and YLP, while the left nanopillar shows both low transmission for both polarizations.

S2. Theoretical analysis of dual-channel design at wavelength of 633 nm

When incident light is polarized parallel to the long or short axis of an unrotated rectangular nanopillar, the transmitted light does not have a cross-polarization component. Therefore, the transmission Jones matrix M_0 can be described as:

$$M_0 = \begin{bmatrix} A & 0 \\ 0 & B \end{bmatrix} \tag{S1}$$

where A and B are the complex transmission (or reflection) coefficients of the polarized light along the x- and y-axes, respectively. When the nanopillar is rotated by an in-plane orientation angle of α, the matrix M_0, after coordinate transformation, can be represented as the new Jones matrix M_α:

$$\begin{aligned} M_\alpha = R(\alpha)\cdot M_0 \cdot R(-\alpha) &= \begin{bmatrix} \cos\alpha & -\sin\alpha \\ \sin\alpha & \cos\alpha \end{bmatrix}\begin{bmatrix} A & 0 \\ 0 & B \end{bmatrix}\begin{bmatrix} \cos\alpha & \sin\alpha \\ -\sin\alpha & \cos\alpha \end{bmatrix} \\ &= \begin{bmatrix} A\cos^2\alpha + B\sin^2\alpha & (A-B)\cos\alpha\sin\alpha \\ (A-B)\cos\alpha\sin\alpha & A\sin^2\alpha + B\cos^2\alpha \end{bmatrix} \end{aligned} \tag{S2}$$

where R(α) is the rotation matrix. Due to the close relationship between the Jones matrix and the input and output fields, a polarizer and an analyzer are employed to select the polarization component of the incident and transmitted light in our design. Upon incidence of a linearly polarized light with polarization angle θ_1 (Jones vector J_0), the transmitted light transmitting through the metasurface (M_α) and passes passing through the analyzer (M_θ) and the Jones vector J_1 of the final outgoing light can be finally expressed as:

$$\begin{aligned} J_1 = M_\theta \cdot M_\alpha \cdot J_0 &= \begin{bmatrix} \cos^2\theta_2 & \sin\theta_2\cos\theta_2 \\ \sin\theta_2\cos\theta_2 & \sin^2\theta_2 \end{bmatrix} \\ &= \begin{bmatrix} \cos^2\theta_2 & \sin\theta_2\cos\theta_2 \\ \sin\theta_2\cos\theta_2 & \sin^2\theta_2 \end{bmatrix}\begin{bmatrix} A\cos^2\alpha + B\sin^2\alpha & (A-B)\cos\alpha\sin\alpha \\ (A-B)\cos\alpha\sin\alpha & A\sin^2\alpha + B\cos^2\alpha \end{bmatrix}\begin{bmatrix} \cos\theta_1 \\ \sin\theta_1 \end{bmatrix} \\ &= \begin{bmatrix} \frac{1}{2}\left((A-B)\cos(2\alpha-\theta_2-\theta_1) + (A+B)\cos(\theta_2-\theta_1)\right)\cos\theta_2 \\ \frac{1}{2}\left((A-B)\cos(2\alpha-\theta_2-\theta_1) + (A+B)\cos(\theta_2-\theta_1)\right)\sin\theta_2 \end{bmatrix} \end{aligned} \tag{S3}$$

where M_θ is the Jones matrix of the analyzer, θ_1 and θ_2 are the transmission axis directions of the polarizer and analyzer, respectively. Given that the intensity of the linearly polarized light after the polarizer is I_0, the intensity of the light passing through the final analyzer becomes:

$$I_1 = I_0\left[\frac{A-B}{2}\cos(2\alpha-\theta_2-\theta_1) + \frac{A+B}{2}\cos(\theta_2-\theta_1)\right]^2 \tag{S4}$$

For an ideal polarizer (i.e. A = 1, B = 0), then Equation (S4) can be simplified as:

$$I_1 = \frac{1}{4}I_0[\cos(2\alpha-\theta_2-\theta_1) + \cos(\theta_2-\theta_1)]^2 \tag{S5}$$

S3. Performance analysis of the metasurface in a broadband spectrum

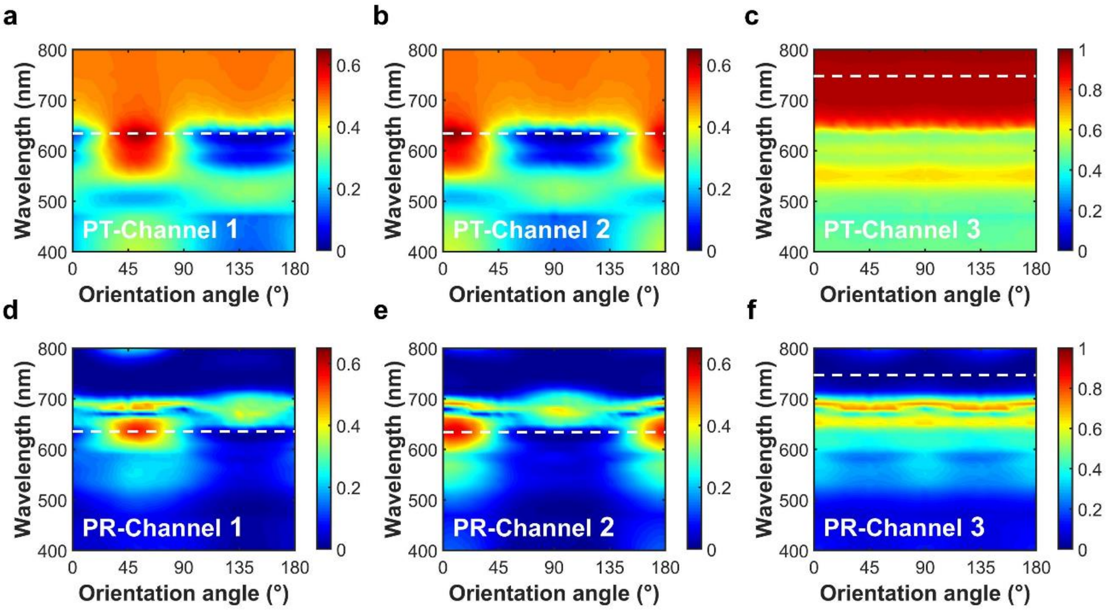

Figure S2. Simulated transmission as a function of the orientation angle of PT in (a) Channel 1, (b) Channel 2 and (c) Channel 3 across a broadband spectrum. Simulated transmission as a function of the orientation angle of PR in (d) Channel 1, (e) Channel 2 and (f) Channel 3 across a broadband spectrum.

The proposed metasurface's ability to operate over a broad wavelength range depends on whether the pre-designed three-channel meta-images can be achieved at other wavelengths. Since the realization of the three-channel meta-images is based on the orientation angle of the two selected nanostructures of PT and PR, we numerically investigated the three-channel transmission profiles as a function of nanostructures' orientation angle, and the relevant results are shown in Figures S2(a)-S2(f). For the PT, the desired transmission profiles in channels 1 and 2 are achieved within the wavelength range of 570-640 nm, while channel 3 maintains high transmission stability from 650 nm to beyond 800 nm. In contrast, for the PR, the transmission in channel 3 remains consistently low, enabling broadband meta-image formation in this channel. However, for channels 1 and 2 of the PR, the desired transmission profiles are only achievable within the narrower range of 600-640 nm. The mismatch in the working wavelength ranges of the PT and PR for channels 1 and 2 ultimately limits the metasurface's ability to operate across a broadband spectrum.

S4. Measured transmission spectra (400-800 nm) of the PT and PR samples under XLP and YLP incidences

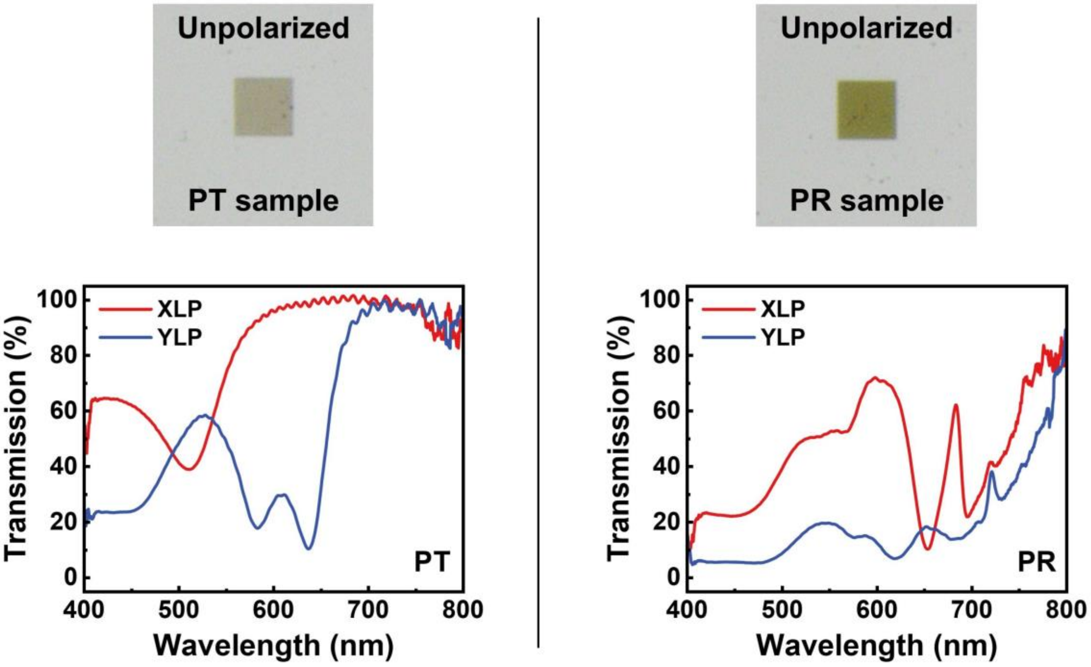

Figure S3. Microscopic images of the PT and PR samples under unpolarized white light and their corresponding measured transmission spectra (400-800 nm) under incidence of XLP and YLP.

Two metasurface samples (denoted as PT sample and PR sample), with each occupying an area of 32×32 μm^2 containing only non-rotated PT and PR, are used to find the two practical operating wavelengths. Two microscopic images of the PT and PR samples captured under unpolarized white light are provided. The measured polarized transmission spectra (400-800 nm) are shown in Figure S3. For the fabricated PT and PR samples, their selected operating wavelengths are 610 nm and 700 nm.

S5. Numerical simulation settings for the three-channel metasurface

In the simulation of the three-channel metasurface, perfectly matched layer boundary conditions are applied in the x, y, and z directions. A plane wave source is used, and a "Frequency-domain field profile" monitor is placed in the x-y plane which is 80 nm above the metasurface, to obtain the near-field information. The near-field images, displayed in terms of the amplitude of the electric field, are directly obtained from the monitor.

S6. Simulated light intensity distributions from the metasurface under various incident polarizations at the wavelength of 750 nm

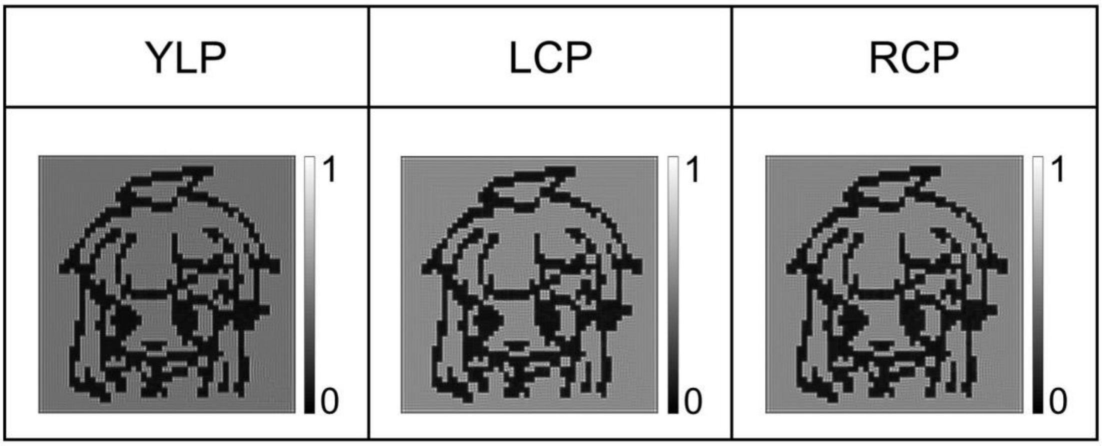

Figure S4. Simulation results obtained under light incidence of different polarizations at the wavelength of 750 nm.

Figure S4 presents the simulated transmitted light intensity distributions from the metasurface, under the incidence of linearly polarized light along the y-axis (YLP), left circular-handed polarized light (LCP), and right circular-handed polarized light (RCP).

S7. Analysis of crosstalk level

Regarding the analysis of crosstalk level, we use the correlation coefficient (*CC*) to judge whether the meta-image is as desired or not. The *CC* is used to calculate the level of similarity between the target image and the experimental image [1]. The computational formula is given in equation (S6):

$$CC = \left(I_{\text{target}}, I_{\text{experiment}}\right) = \frac{Cov(I_{\text{target,}} I_{\text{experiment}})}{\sqrt{Cov(I_{\text{target,}} I_{\text{target}})}\sqrt{Cov(I_{\text{experiment}}, I_{\text{experiment}})}} \tag{S6}$$

where I_{target} and $I_{\text{experiment}}$ represent the intensity values of the target image and the experimental image, respectively, while *Cov* represents the covariance operation. A *CC* value of 1 indicates perfect positive correlation, meaning the two images are identical with no crosstalk. Typically, a *CC* value larger than 0.6 indicates the two images are strongly correlated with low crosstalk. We respectively calculated the *CC* values between each experimental image and the target images from different channels, as shown in Table 1. For Meta-images 1, 2, and 3, the *CC* values with their corresponding target images are 0.88, 0.93, and 0.90, respectively. In contrary, the *CC* values in different channels are significantly lower than these values. Overall, the results suggest that the experimentally obtained images agree very well with the target images, with negligible crosstalk.

Table 1. *CC* of target images and experimental images between different channels

CC	Meta-image 1 (experiment)	Meta-image 2 (experiment)	Meta-image 3 (experiment)
Meta-image 1 (Target)	0.88	0.19	0.12
Meta-image 2 (Target)	0.10	0.93	0.17
Meta-image 3 (Target)	0.19	0.25	0.90

S8. Metasurface B and C are used to hide textual information

S8.1 Designed metasurface B and C

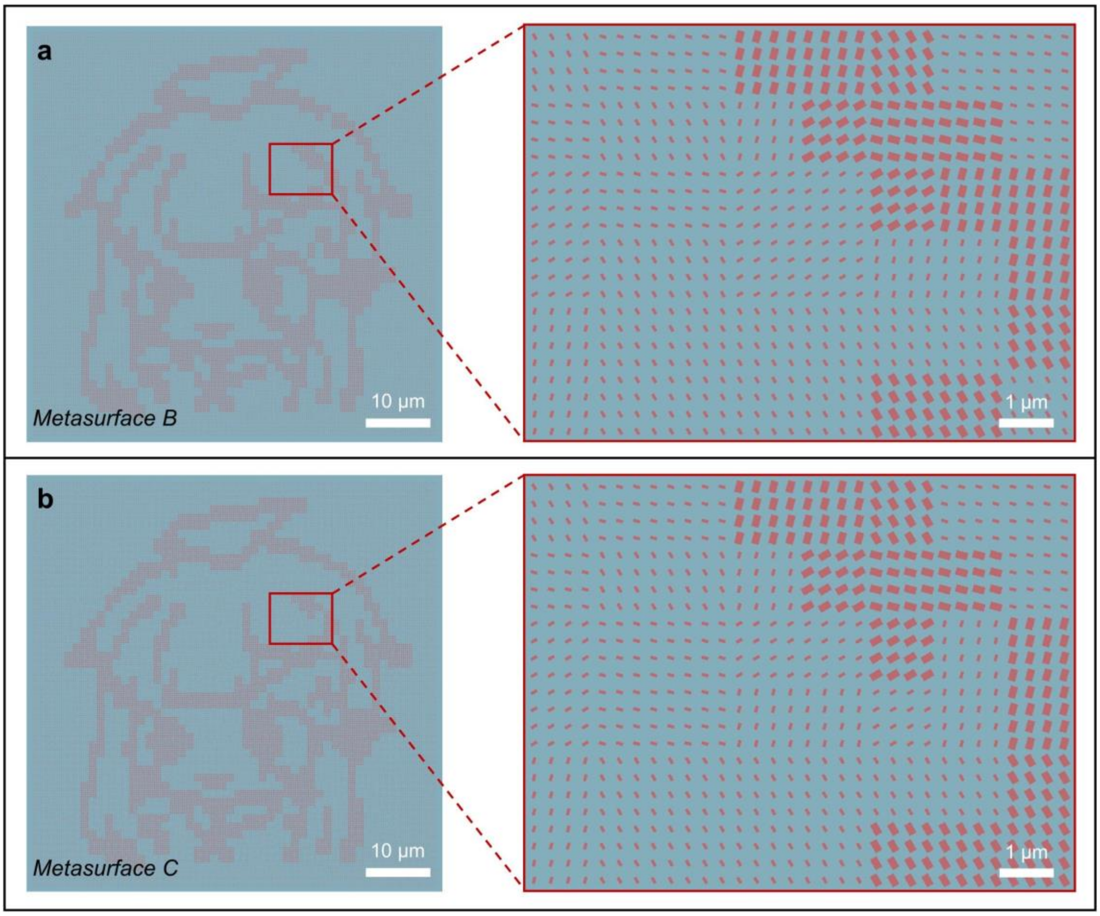

Figure S5. (a) Appearance image of metasurface B. (b) Appearance image of metasurface C.

Here, metasurfaces B and C are designed to hide the textual information. Both metasurfaces are designed to display the same carrier images, whereas they convey very different secret information written in Chinese and English, respectively. We present the overall and partial appearance images of metasurface B and C, as shown in Figures S5(a) and S5(b), respectively. It can be observed that there are differences in the nanopillar types and their orientation angles. These differences are precisely caused by the different hidden information.

S8.2 Embedding and extraction process of textual information

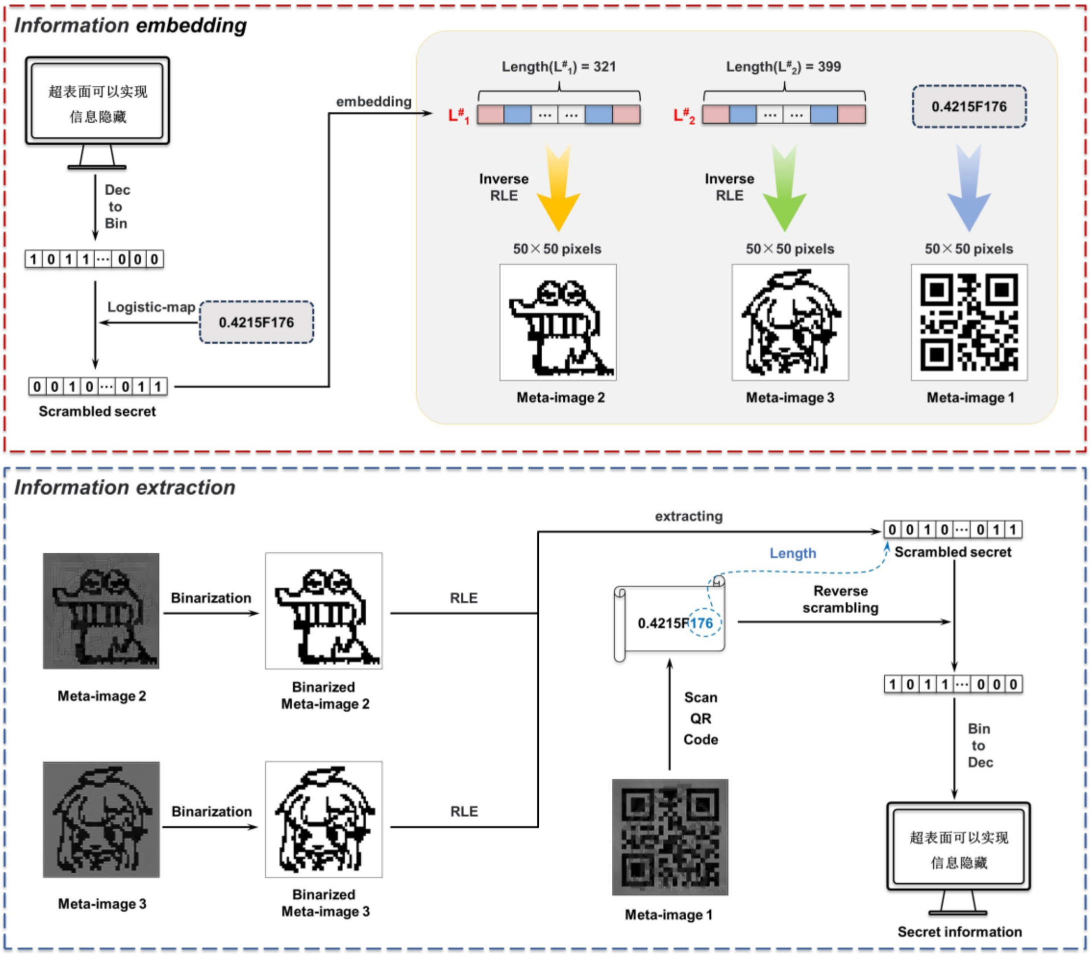

Figure S6. Embedding and extraction process of information in metasurface B.

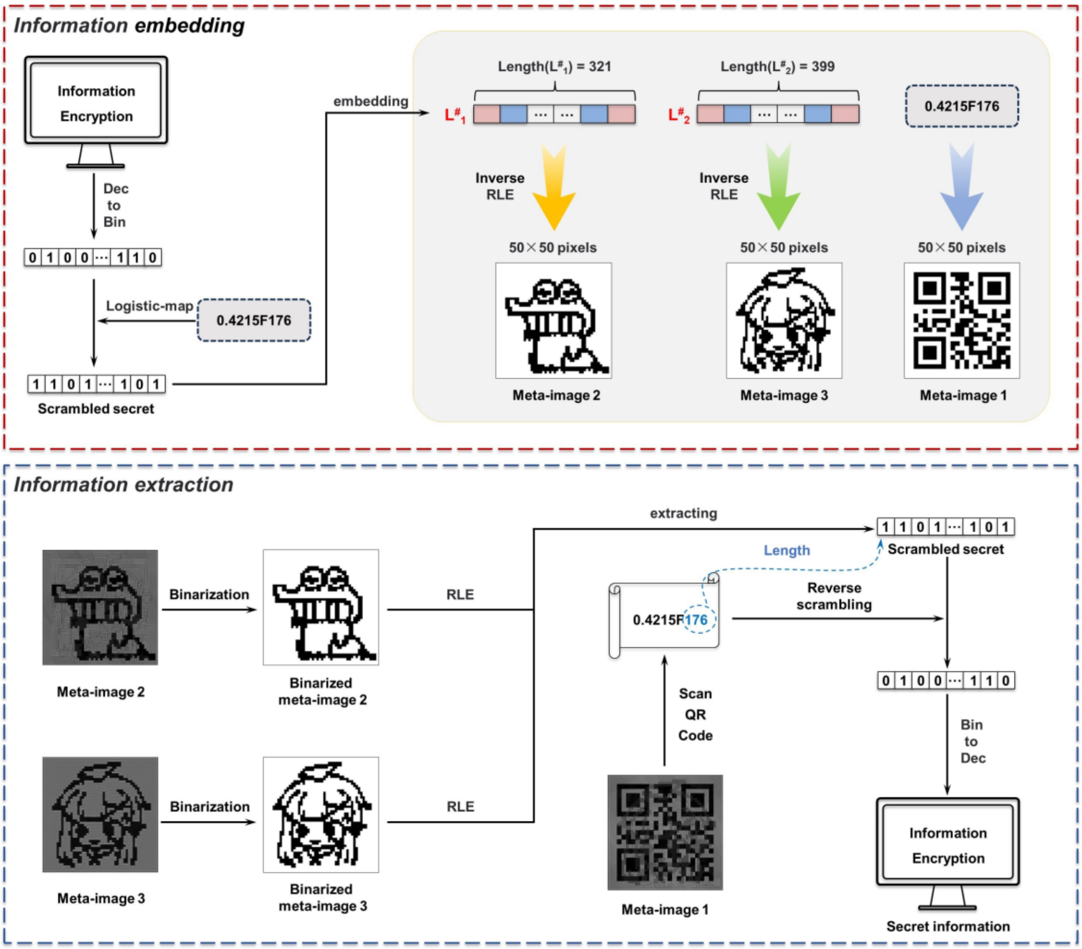

Figure S7. Embedding and extraction process of information in metasurface C.

Here, we provide the embedding and extraction processes of the secret textual information in metasurface B and C, respectively, as illustrated in Figures S6 and S7. Unlike image information hiding, the main difference lies in the conversion between decimal and binary for secret textual information. The parameter 0.4215 and 176 in the parameter 0.4215F176 represent the initial value of the logistic map and the length of embedded data, respectively. The letter F is used to distinguish between the two values and holds no actual significance.

S9. Error analysis in information extraction and recovery process

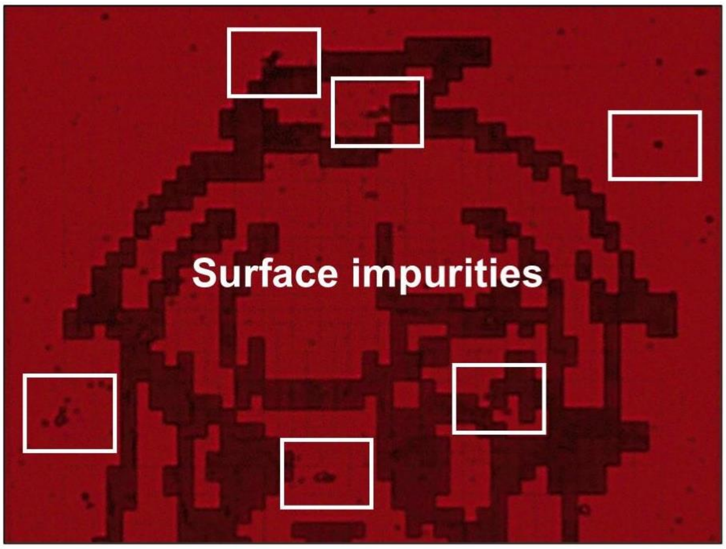

Figure S8. Local meta-image showing surface impurities. The white boxes highlight the defect area.

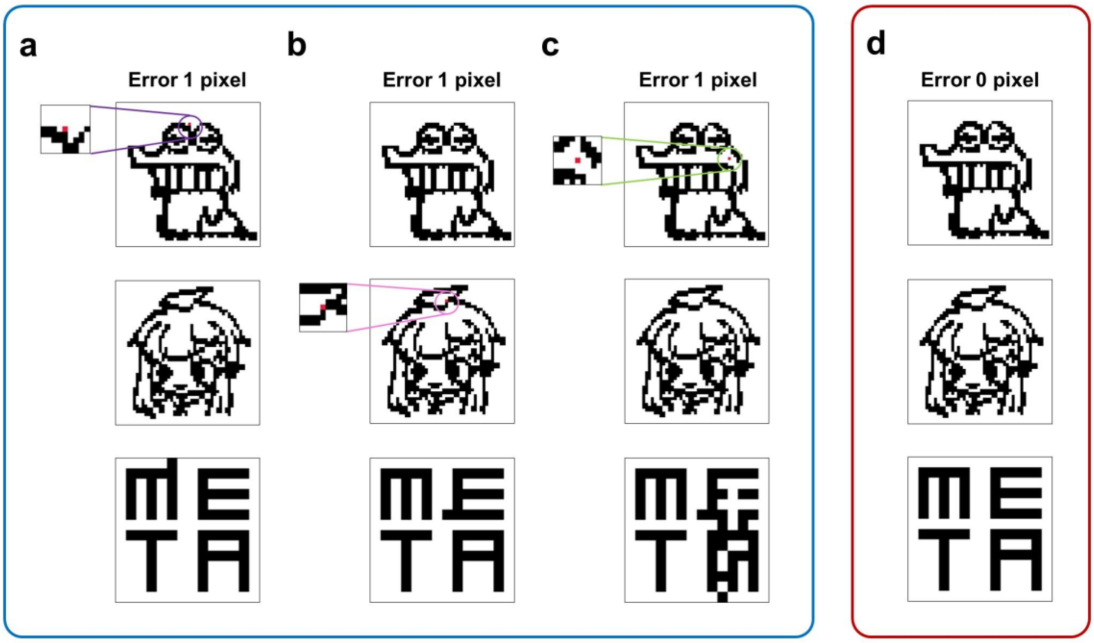

Figure S9. Error analysis of the decryption process. (a-c) Different errors appeared in the pixels of the meta-images. (d) The secret information obtained when the meta-images are accurately obtained. The red pixels indicate where the error occurs.

The extraction of the secret information relies on the identification of pixels in the meta-images. Here, the possible errors in the process of secret information extraction and recovery are mainly caused by surface impurities (Figure S8). When the impurity on the sample surface is too serious, it will cause the pixels in the meta-images to change. Since run-length encoding is a lossless compression encoding, eventually these changes will be reflected in the secret

information. As a simulated demonstration, we manually introduced one erroneous pixel into the binarized meta-images and extracted the secret information from the erroneous images. When the error pixel appears at the alternating boundaries of black and white pixels in each column of the binarized meta-images, it will only affect the parity of the corresponding run-length value of the pixel position, and then make the corresponding pixel in the secret information wrong, as shown in Figures S9(a) and S9(b). However, when the error pixel occurs in the non-boundary position, it may affect the run-length values of all subsequent information extraction locations, which will then have a serious impact on the secret information, as shown in Figure S9(c). It is worth noting that a small amount of data destruction in meta-images could result in a large number of errors in retrieved information, and this pixel-sensitive method of information hiding can effectively avoid brute force attacks and facilitate the destruction of encrypted information in secure transmission applications. In the decryption process, since these impurities on the sample surface is slight, and the light and dark contrast of the image obtained in the experiment is relatively uniform, we use the global threshold and nearest interpolation to process the experimental images, which can avoid the effect of existing impurities on the surface of this sample, so that the secret information can be accurately extracted, shown in Figure S9(d).

S10. Discussion on further increasing information channels and security

The increase in information channels can be viewed in terms of both the number of meta-image channels and the amount of secret information. In the current case, a one-to-three mapping strategy is employed between the secret information and the meta-image channels. This mapping strategy is determined by the whole process of embedding the secret information in the carrier meta-images, including the RLE, information preprocessing and the information embedding processes. Therefore, without modifying the whole processes, adding one additional secret information requires three extra meta-image channels. Thus, it becomes necessary to come up with a metasurface to accommodate *3n-channel* near-field meta-images when hiding *n* secret information, which may demand metasurface design strategies such as the noise-engineered metasurface [2] or spatially multiplexed metasurfaces. Surely speaking, the rigorous one-to-three mapping strategy can be broken down by proposing a new whole process, which could be one of the focuses in our future work. Additionally, by incorporating dynamic control mechanisms such as electrical, optical, or thermal tuning, metasurfaces can achieve multifunctional switching under different temporal or conditional scenarios, thereby significantly improving the flexibility and diversity of information processing. Some electrically-tuned metasurfaces can also be utilized in our current work to increase the complexity for information decryption. For instance, the proposed three-channel binary encoding can be set under different biasing voltages, so that in addition to the predesigned optical keys, these meta-images are only visible under the correct voltages.

S11. Quantitative analysis of encryption complexity and relevant work comparisons

The encryption complexity (information depth) can be quantitatively analyzed from the perspective of information entropy or Shannon entropy. According to the information theory proposed by Shannon, higher the entropy represents more uncertainty in the information, making the information more complicated. Therefore, the information entropy can be used to quantitatively analyze the complexity of information encryption during the process of embedding secret information. The formula for calculating information entropy is:

$$H(X) = -\sum_{i=1}^{i=Max\ (D)} p(x_i) \log_2 p(x_i) \tag{S7}$$

where X represents the embedding process of secret information, $p(x_i)$ is the probability of embedding the *i-th* binary number into a specific carrier image (*Max (D)* represents the length of information). When binary numbers in an information sequence are embedded in two run-length sequences according to rules, $p(x_i) = 0.5$. When embedded only in one run-length sequence, $p(x_i) = 1$. After calculation, the information entropy is 160 bits. Compared with the reference works [33-35, 49, 50] in the main text, most of them remain a form of pure optical encryption, where secret information is directly obtained by switching optical components, and the encrypted pieces of information are independent of each other. The similarity between the proposed work and these studies lies in the design of multi-channel metasurfaces to store multiple meta-images, introducing comparable complexity in terms of meta-image decryption; nevertheless, the roles of these meta-images differ significantly. In contrast to the methods that directly retrieve secret information through a single optical encryption approach, the key significance of the proposed work is that it requires not only optical decryption but also subsequent algorithmic decryption, which is believed to have substantially enhanced the security of the secret information.

S12. Experimental setup

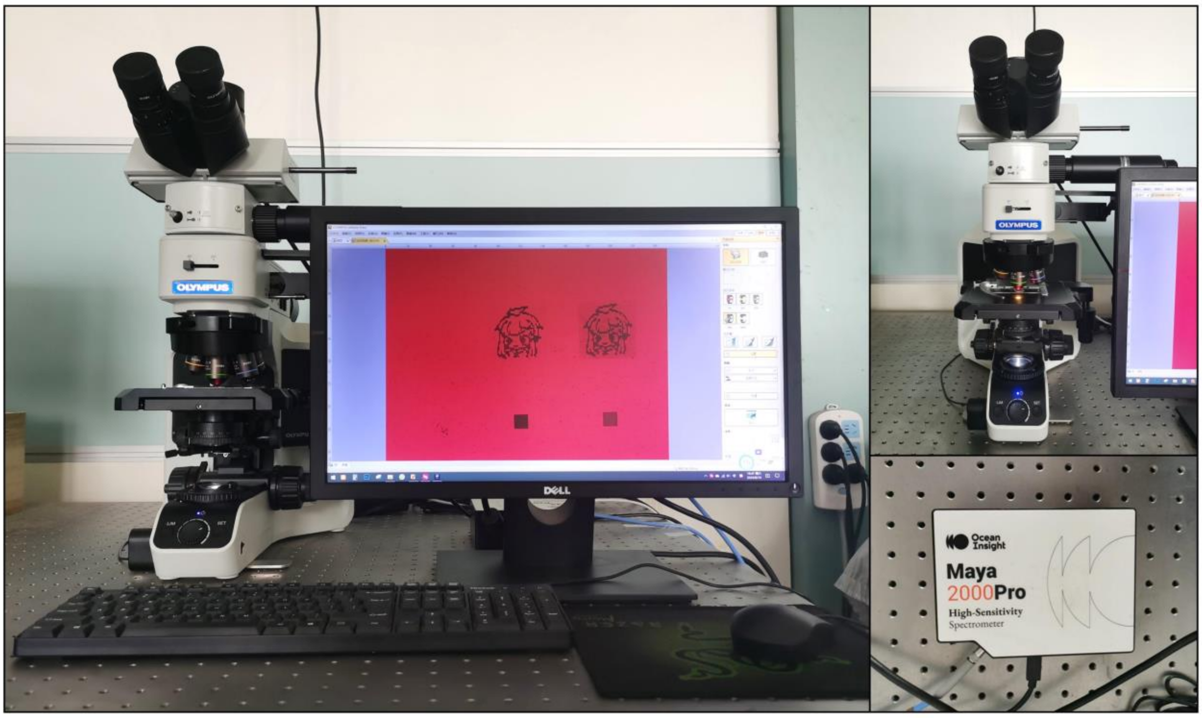

Figure S10. The experimental setup for capturing the image and measuring the transmission spectra of the samples.

Reference

[1] J. Deng, F. Gao, P. Yuan, Y. Li, and B. Yan, “Bidirectional nanoprinting based on bilayer metasurfaces,” *Opt. Express*, vol. 30, no. 1, pp. 377-388, 2022.

[2] B. Xiong, et al., “Breaking the limitation of polarization multiplexing in optical metasurfaces with engineered noise,” *Science*, vol. 379, no. 6629, pp. 294-299, 2023.
